# A computational framework for epigenetic plasticity in memory

**DOI:** 10.1093/brain/awag094

**Published:** 2026-03-07

**Authors:** Geoffroy Delamare, Surbhit Wagle, Johannes Gräff, Claudia Clopath

**Affiliations:** Bioengineering Department, Imperial College London, London SW7 2AZ, UK; Bioengineering Department, Imperial College London, London SW7 2AZ, UK; Laboratory of Neuroepigenetics, Brain Mind Institute, School of Life Sciences, Ecole Polytechnique Fédérale de Lausanne, Lausanne 1015, Switzerland; Bioengineering Department, Imperial College London, London SW7 2AZ, UK

**Keywords:** recurrent network, synaptic plasticity, epigenetic plasticity, memory, priming, allocation

## Abstract

Memories are thought to be encoded in synaptic connections between assemblies of neurons that are reactivated during memory recall. However, in light of ongoing molecular turnover and synaptic decay, this widely accepted view cannot explain how individual ensembles are maintained over (life-)long timescales. Experimentally, learning has not only been associated with synaptic modifications among neurons, but also with epigenetic alterations of learning-related gene transcription within neurons. Although these epigenetic changes are involved in all stages of memory dynamics, they have been largely omitted in computational studies.

In this update, we advocate for the integration of epigenetic mechanisms into computational models of memory. Using a recurrent neural network model that includes epigenetic plasticity as a variable, we explore the role of epigenetic priming in the maintenance of memories across long timescales; we then investigate the implications of epigenetic modifications for memory allocation and for reversing cognitive decline associated with neurodegeneration; and finally, we predict several computational advantages of including epigenetics over traditional models of synaptic memory.

Overall, this paper stands as a first step towards the integration of epigenetics in computational models of memory and corroborates the experimentally derived notion that memory might not be encoded solely in synaptic weights, but rather co-encoded in epigenetic patterns within the nucleus.

## Teaser

Delamare *et al.* present a computational model of memory formation inspired by recent experimental data, in which individual memories are encoded not only in synapses, but also in the nucleus via epigenetic plasticity.

## Introduction

Understanding memory circuits in the brain requires two fundamental questions to be answered. How can memories be rapidly encoded, yet be maintained stably over timescales that can reach a lifetime? The main neuroscientific approach to answer these questions has considered that information is encoded at the synaptic level. Indeed, upon learning, rapid changes among synapses give rise to specific connections among neurons. The corresponding activity patterns are reactivated when memory is retrieved, resulting in a neural code that is correlated with memory read-out.^1^ Correspondingly, most computational models of memory assume that information is encoded within synapses. In particular, Hebbian learning rules can successfully reproduce their long-term potentiation (LTP),^2,3^ and memories can be modelled as neural ensembles formed through Hebbian associations, which are stable across time^4-7^ and able to perform pattern separation and completion, making them particularly powerful to model memory engrams.^8,9^ Nevertheless, such models also require that synapses are maintained over long timescales for memory maintenance, although no experiment to date has directly proved learning-induced synaptic stability over extended timescales (but see also Abraham *et al*.^10^ for high-frequency stimulation-induced long-lasting LTP). This last point thus represents a fundamental stability problem. If long-term memories are maintained solely in synapses, how can they be resilient to synaptic and protein turnover that happen at much shorter timescales?^11,12^ Only few synaptic-based models of memory circuits have tackled this question so far,^8,13-15^ and memory models have been largely agnostic to cell-intrinsic changes within individual neurons.

In the past two decades, several experimental studies have found that learning is associated with changes to the epigenetic state of a neuron.^16-19^ Similar to cellular memories,^20^ for which epigenetic modifications stably dictate changes in gene expression during developmental differentiation,^21^ epigenetic mechanisms have been proposed to maintain behavioural memories over long timescales.^19,22,23^ Indeed, epigenetic mechanisms are implicated in all phases of a memory, from its encoding,^24^ consolidation^25^ and retrieval^26^ to its extinction.^27^ Their role has been associated with learning-induced increases in the transcription of genes related to synaptic and intrinsic plasticity, thereby permitting a bidirectional dialogue between synapses and genes. However, computational models have thus far omitted the role of epigenetics in memory dynamics. Here, we propose a computational model of memory that integrates epigenetic plasticity in the face of synaptic decay.

## Materials and methods

### Recurrent neural network with excitability

Our network is composed of *N* = 50 neurons, with firing rate *r_i_*, 1 ≤ *i* ≤ *N*, that follow a rate-based dynamics given by:

(1) τrdridt+ri=ReLU(Δi(k)(t)+∑j=1NWijrj−I+εi(t))

where *τ_r_* = 20 is the decay time of the rates and ReLU is the rectified linear activation function. Recurrent connections, *W*, are all-to-all and plastic, and follow a Hebbian rule given by:

(2) $$\frac{dW_{ij}}{dt}=\frac{r_{i}r_{j}}{\tau_{i}^{+}(t)}-\frac{W_{ij}}{\tau_{-}}$$

where *i* and *j* correspond to the pre- and postsynaptic neurons, respectively, and where $\tau_{i}^{+}(t)$ and *τ*^−^ = 4000 are the learning and the decay time constants of the weights, respectively. The dynamics of $\tau_{i}^{+}(t)$ are described in the next section. A hard bound of [0,1] was applied to these weights. Inhibition, *I*, is given by:

(3) $$I=I_{0}+I_{1}\overset{N}{\underset{i=1}{\sum }}\;r_{i}+I_{2}\overset{N}{\underset{i=1}{\sum }}\;r_{i}^{2}$$

where *I*_0_ = 7, *I*_1_ = 0.5 and *I*_2_ = 0.05. Δ^(*k*)^(*t*) denotes the input from context *k* ∈placeholder, with dynamics explained in the protocol section below. Finally, excitability is modelled as a time-varying threshold, *ε_i_*(*t*), of the input–output function of each neuron *i*, with dynamics described in the next section.

### Epigenetic priming

All neurons *i* are attributed a priming variable *α_i_*, which is initially set to zero. During encoding, if a neuron *i* reaches the firing threshold of *θ* = 2, the priming variable rapidly increases to two over a timescale of $\tau_{\alpha }^{+}=10$, for a duration of 100 time steps:

(4) $$\frac{d\alpha_{i}}{dt}=\frac{2-\alpha_{i}}{\tau_{\alpha }^{+}}$$

Then, this priming variable decays to its steady state value of one, over a timescale of $\tau_{\alpha }^{-}=1000$ for the rest of the simulation:

(5) $$\frac{d\alpha_{i}}{dt}=\frac{1-\alpha_{i}}{\tau_{\alpha }^{-}}$$

Finally, we assume that intrinsic excitability and learning rate are varying based on this epigenetic modification. In our model, the learning time constant of the Hebbian rule $\tau_{i}^{+}$ and excitability $\varepsilon_{i}^{+}$ of neuron *i* are subject to the following dynamics:

(6) $$\varepsilon_{i}^{+}(t)=\varepsilon_{i}^{0}(1+\beta_{\varepsilon }\alpha_{i}(t)),\;\mathrm{if}\;r_{i}>\theta_{\alpha }$$

(7) $$\tau_{i}^{+}(t)=\frac{\tau_{0}^{+}}{1+\beta_{\tau }\alpha_{i}(t)},\;\mathrm{if}\;r_{i}>\theta_{\alpha }$$

where $\tau_{0}^{+}=2500$ and where $\varepsilon_{i}^{0}$ is sampled randomly from a χ^2^ distribution of parameter 1 at each time step. When excitability is increased concurrently with epigenetic priming, 0.1 is added to $\varepsilon_{i}^{+}(t)$, for every primed neuron *i*, during memory encoding. Note that transcription happens only if neurons reach the firing rate, *θ_α_*. Parameters *β_W_* and *β_ε_* are scaling factors that are tuned for down- or upregulation of epigenetics as follows: *β_W_* = 0.9 (control), 0.8 (downregulation) and 1.0 (upregulation); and *β_ε_* = 0.9 (control), 0.8 (downregulation) and 1.0 (upregulation).

### Simulation protocol

We consider that the memory is initially encoded and later reactivated during memory recall, at different time points. The encoding stimulation consists of 10 repetitions of interval 100 time steps (ts), spaced by a inter-repetition delay of 100 ts. A random half of the neurons correspond to the first context. The second context corresponds to 95% of the other half and 5% of the neurons from the first context. When (*k*) a context is presented, Δ*_i_* (*t*) takes the value *δ* = 16 if *i* belongs to the corresponding context ensemble, and *δ/*10 otherwise. The first context and second one (if any) are presented at *t* = 1000 and *t* = 4000. Recall consists of a single stimulation of duration of 100 ts, starting at *t* = 8000 and *t* = 11 000 for the first and second context, respectively. Recall is repeated four times, with an inter-repetition delay of duration of 6000 ts. The overlap between two ensembles was computed as the number of neurons being active during presentation of both contexts, divided by the number of neurons active during the first one. It was computed during the last repetition of the encoding stimulation (encoding overlap) and during the last recall stimulation (recall overlap).

### Modelling neurobiological mechanisms interfering with memory

We induced memory loss attributable to decreased maximum synaptic capacity by setting a lower hard bound of 0.85 on the maximum synaptic weights (*W*). For increasing the synaptic decay rate, we set *τ*^−^ = 3800. To model synaptic noise, we added Gaussian noise with mean zero and standard deviation 0.001 to synaptic weight update at each time step. Finally, we modelled memory loss attributable to synaptic loss. For this, after encoding (i.e. *t* = 3000), we pruned synapses whose value went below a pruning threshold of 0.2 with a probability of 30%. We applied synaptic pruning after every 1000 time steps. Once a synapse was pruned, its value was set to zero for the rest of the stimulation.

### Model of synaptic tag and capture

We simulated synaptic tag and capture mechanisms in our model by using two different decay time constants, *τ*^−^ = 3500 or *τ*^−^ = 4300, to model the early- and late-phase LTP. Initially, all synapses (*W*) were in an untagged state, in which they decayed faster with the time constant of *τ*^−^ = 3500. During the simulation, if the synaptic weight change within a single step grew above the threshold of 0.003 (i.e. Δ*W_i_*_,*j*_ > 0.003, reflecting strong Hebbian synaptic plasticity), the corresponding synapse was set to a tagged state, in which it decayed with a slower decay constant of *τ*^−^ = 4300 for the rest of the simulation time.

### Cognitive deficiency

Cognitive deficiency is applied by decreasing the learning rate, $\tau_{0}^{+}=2800$. Enhanced epigenetics was applied by increasing *β_W_* and *β_ε_* to 1.5.

## Results

### Synaptic instability leads to loss of memory

**Figure 1:**
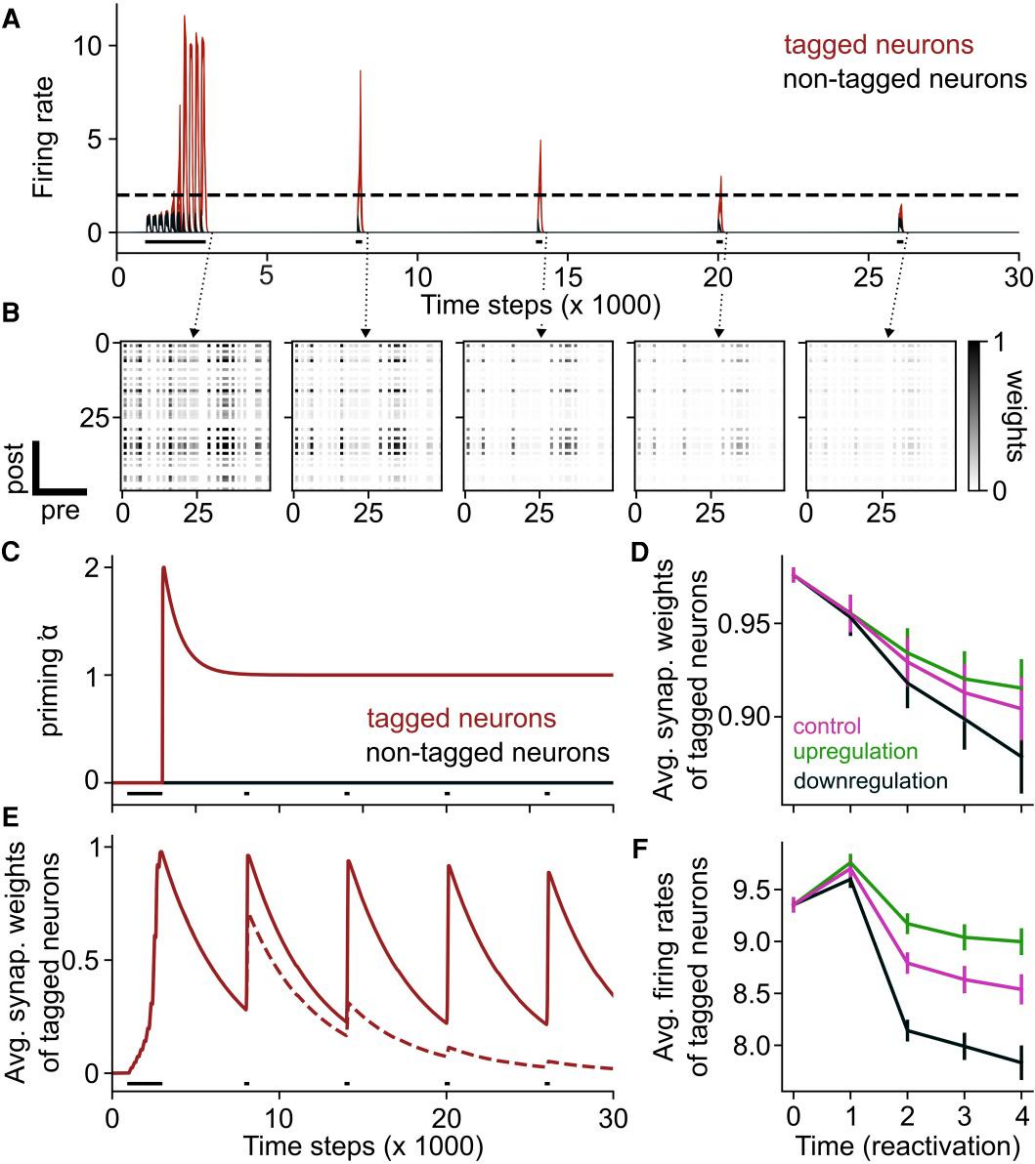
**Epigenetic mechanisms can compensate for memory loss associated with synaptic decay.** (**A**) Firing rates of all neurons across time. Neurons tagged during encoding are shown in red and other neurons in black. Black bars show stimulation of the network, with the first one corresponding to encoding and the last four to recall. The dashed line corresponds to the active threshold. (**B**) Synaptic weights right after encoding (*first panel*) and immediately after each of the four reactivations (*second* to *fifth panels*). (**C**) Dynamics of the priming variable. Tagged neurons are shown in red and non-tagged neurons in black. The priming variable of tagged neurons increases to two after encoding before decreasing to one, its steady-state value. (**D**) Recurrent synaptic weights of tagged neurons in three different scenarios: when epigenetics is kept at baseline (magenta); downregulated, thereby preventing transcription (black); or upregulated, thereby increasing transcription (green). (**E**) Averaged synaptic weights of tagged neurons across time when epigenetic modifications are considered (solid line) and when not (dashed line). Average over *n* = 10 simulations. (**F**) Recurrent firing rates of tagged neurons in three different scenarios: when epigenetics is kept at baseline (magenta); downregulated, thereby preventing transcription (black); or upregulated, thereby increasing transcription (green). Data are shown as the mean ± standard error of the mean, *n* = 100 simulations.

To gain a better understanding of the effect of synaptic decay^12^ on memory maintenance, we have designed a new model of memory encoding and recall using a recurrent neural network. Its synaptic weights follow a Hebbian rule and are subject to decay over a longer timescale (see the ‘Materials and methods’ section). When we initially stimulated our network, we observed the formation of a neural assembly akin to engram cells. During encoding, a subset of neurons has firing rates above the active threshold, and the same subset is reactivated during recall (Fig. 1A). At the synaptic level, neurons from these subsets are connected recurrently (Fig. 1B).

We also observed that although subsequent reactivations of the ensemble induced an increase of synaptic weights, they were insufficient to maintain the ensemble across time (Fig. 1A and B). Indeed, firing rates of the tagged neurons decreased below the active threshold after a few reactivations. Therefore, we may now ask, how can memory be maintained across time despite synaptic decay?

### Epigenetic priming of tagged neurons improves memory retention

We hypothesized that following encoding, intrinsic changes to the epigenome of individual neurons could support memory maintenance. This hypothesis is based on findings by us and others that learning is accompanied by epigenetic changes^24,28,29^ and that manipulating the epigenome within specific cell populations can, in turn, alter mnemonic capacities.^30,31^

In our model, we introduced epigenetic regulation of two neural factors: intrinsic excitability (IE) and learning rate. During encoding, each neuron is attributed with an epigenetic priming variable. If the neuron is active during encoding, this variable is set to increase, before decreasing to a steady value (Fig. 1C), akin to observations *in vivo*.^26,32^ Neurons in this state are now ‘primed’ and ready to trigger plasticity-related gene transcription following neural activity, in particular during the successive reactivations of the ensemble. With this configuration, increases of IE and learning rate happen only if a given neuron is primed and if its firing rate is above a given threshold, *θ_α_*.

We observed that the recurrent weights of neurons tagged during encoding were maintained for longer following the introduction of the epigenetic priming variable (Fig. 1D). In addition, epigenetic priming also led to memory stabilization against naturally occurring memory interferences, such as decreased maximum synaptic weights, increased synaptic decay rate, synaptic noise and pruning of weak synapses (Supplementary Fig. 1). Furthermore, to model early- and late-phase LTP, we also incorporated a synaptic tagging and capture (STC) stimulation. In the STC model, synapses that underwent stronger Hebbian learning were tagged and decayed more slowly (to mimic late-phase LTP), whereas untagged synapses decayed faster. Even in this STC model, we observed that epigenetic mechanisms were able to protect against memory loss (Supplementary Fig. 2).

Next, we hypothesized that the manipulation of epigenetic factors that favour chromatin accessibility and therefore the transcription of excitability- and plasticity-related genes (such as histone acetylation, although our model remains agnostic to the specific type of epigenetic modification) would favour the maintenance of synaptic weights,^22^ and vice versa. To test this, we varied the scale to which epigenetic priming affects neuronal parameters such as IE and synaptic plasticity. We found that up- and downregulating epigenetics led to an increase and decrease in both firing rates and synaptic weights of tagged neurons (Fig. 1E and F), respectively.

Taken together, these results indicate that the loss of memories associated with synaptic decay in a neural circuit model can be compensated when taking an epigenetic (transcription-promoting) priming variable into consideration.

### Epigenetics *per se* does not affect memory allocation

We then tested whether epigenetic mechanisms before learning affect memory allocation, the process by which specific neurons in a neural network become recruited to store a given memory.^33^ Initially thought of as stochastic, such neuronal selection into the memory trace does not happen randomly, but depends on the individual state of a neuron, with elevated IE and overexpression of the transcription factor Ca^2+^/cAMP response element-binding protein (CREB) increasing the odds of being allocated into the memory trace.^34,35^

**Figure 2:**
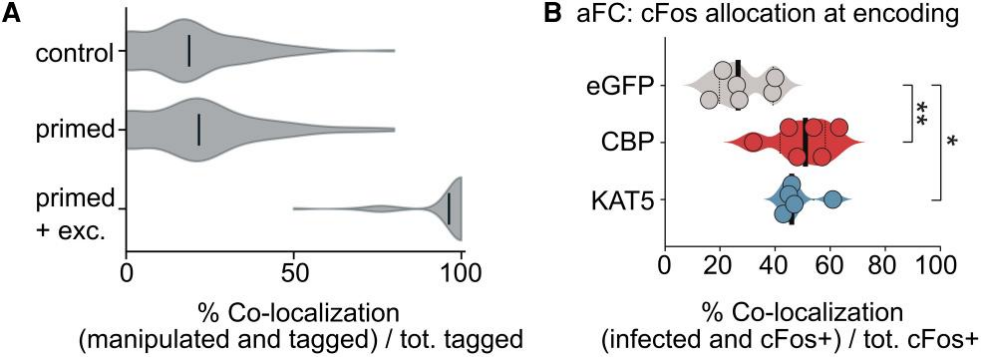
**Epigenetically primed neurons are preferentially allocated to the memory ensemble when their excitability is increased concurrently.** (**A**) Co-localization of neurons in three different scenarios: when no neuron is primed during encoding (control); when a subset of neurons is primed (primed); and when primed neurons also have increased excitability (primed + exc.). *n* = 1000 simulations. (**B**) Figure redrawn from Santoni *et al*.,^36^ showing that neurons with enhanced histone acetylation (mediated by the histone acetyltransferases CBP and KAT5) are preferentially allocated to the memory trace in lateral amygdala. Memory allocation was calculated as the fraction of cFos^+^ neurons that had been infected with eGFP, CBP or KAT5 (e.g. cFos^+^ + CBP^+^) normalized to the total number of cFos^+^ neurons, indicating that the likelihood of being cFos^+^ is higher in the presence of histone hyperacetylation.

Based on our own recent findings,^36^ we posited that 20% of the neurons were already epigenetically primed before memory encoding. During the last encoding stimulation, we then measured the proportion of neurons that both belong to the primed subset and are tagged, i.e. that have had a firing rate above the active threshold during encoding. We found that epigenetic priming *per se* did not alter the proportion of allocated neurons (Fig. 2A).

However, epigenetic priming prior to encoding is cell-intrinsically linked to IE,^36^ prompting us to combine epigenetic priming with IE. Using this approach, we found that most neurons that were primed during encoding were also allocated to the assembly (Fig. 2A), consistent with our experimental data (Fig. 2B).^36^ Overall, therefore, our model confirms the experimentally derived cell-intrinsic interplay between epigenetic mechanisms and IE in memory allocation.

### Epigenetic contribution to neurodegeneration-related cognitive decline

**Figure 3:**
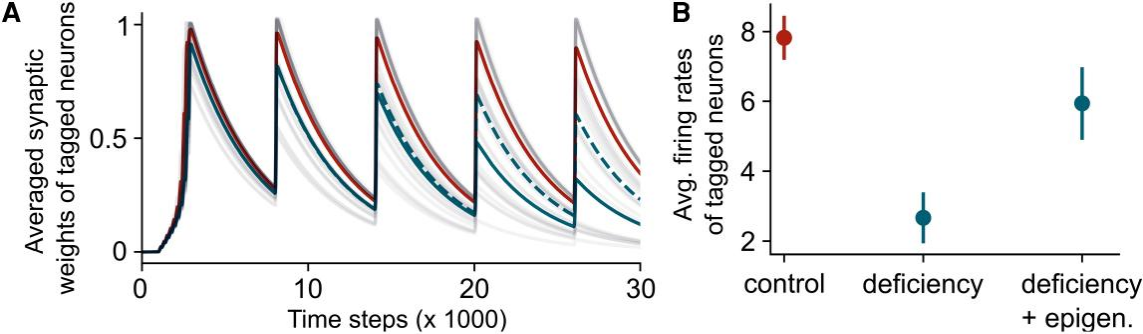
**Increasing epigenetic mechanisms can recover synaptic deficiency.** (**A**) Averaged synaptic weights of tagged neurons across time in the control case with normal epigenetics (red), in the case of deficiency (blue, solid line) and in the case of deficiency with enhanced epigenetics (blue, dotted line). (**B**) Average firing rates of the tagged neurons during the last stimulation (*t* = 26.1) in the three different cases. Data are shown as the mean ± standard error of the mean, *n* = 10 simulations.

Next, we tested whether enhancing epigenetic plasticity could compensate for cognitive decline in neurodegeneration, known to be mediated by impaired synaptic potentiation, slower learning rates and diminished capacities to strengthen synapses,^37-39^ and by epigenetic aberrations preventing synaptic plasticity-related gene expression.^40-43^ We simulated cognitive deficiency by decreasing the learning rate (see the ‘Materials and methods’ section), which mimics working memory impairments^44^ and reduced cognitive flexibility,^45^ thereby isolating the contribution of impaired potentiation without altering forgetting rates. We observed a decrease in synaptic strength (Fig. 3A, solid blue line) leading to reduced memory maintenance (Fig. 3B, solid blue line). Conversely, when we enhanced epigenetic plasticity, as done experimentally by us and others,^40,41,46-48^ we observed a partial recovery of memory loss (Fig. 3A and B, dotted blue line). These findings therefore not only corroborate the empirical evidence that dysregulated epigenetic mechanisms contribute to cognitive decline, but also corroborate computationally that their reversal can potentially rescue it.^49,50^

### Advantages of epigenetics over direct modulation of internal variables

**Figure 4:**
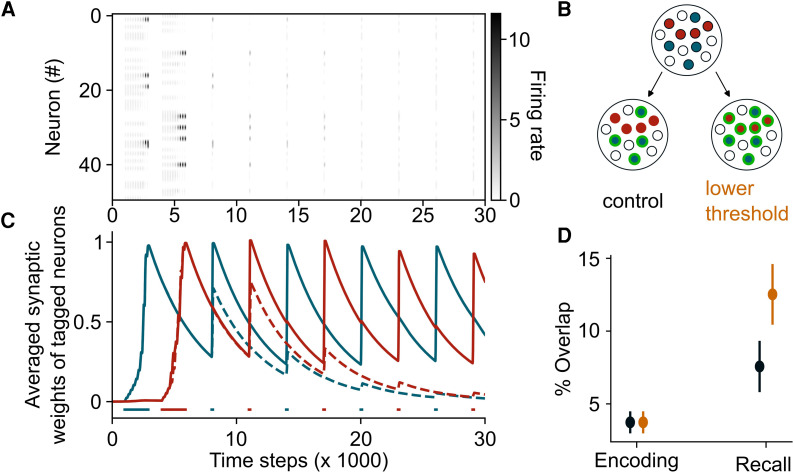
**Epigenetic mechanisms help to consolidate ensembles and to prevent memory interference.** (**A**) Firing rates of neurons across time without epigenetics. Two ensembles are formed by initial stimulation of subsets of neurons (Methods). As in the previous case with a single ensemble, firing rates of neurons from these ensembles decrease across time, indicating memory loss. (**B**) Epigenetics is added in the model in two different ways. Transcription of plasticity- and excitability-related genes increases either when neurons are primed and active during recall (control) or only if they are primed regardless of further activation (lowered threshold). In our model, this corresponds to lowering the threshold for increase of excitability and learning rate. Epigenetic changes are shown in green. (**C**) Averaged synaptic weights of tagged neurons across time when epigenetics is applied (solid line) and absent (dashed line), for neurons tagged in context **A** (red) and in **B** (blue). Average over *n* = 10 simulations. (**D**) Overlap among the two ensembles in these two scenarios, during encoding and recall. Data are shown as the mean ± standard error of the mean, *n* = 100 simulations.

Several experimental^51-53^ and computational^4,54^ studies have shown that increased IE can cause memory interference. As a primarily permissive variable, which comes into play only with further neuronal activation, epigenetic priming might act as a ‘meta-learning’ process, which is silent if neuronal firing rates remain below the activation threshold. An intriguing consequence of this assumption is that it can prevent multiple memories from interfering. To test this hypothesis, we modelled two memories by stimulating different subsets of neurons in an alternate manner (Fig. 4A). We observed the formation of two distinct ensembles that are maintained through epigenetic regulation (Fig. 4B). We then measured their overlap during recall in two conditions: either when primed for reactivation (as above) or when priming was such that the overall firing threshold decreased irrespective of recall (Fig. 4C). We found a higher overlap for ensembles that had an overall reduced firing threshold, independent of recall-induced neuronal activity (Fig. 4D, recall). In other words, silent epigenetic priming allows for reinforcing ensembles independently, while protecting them from interference.

## Discussion

Overall, our study highlights computationally that epigenetic mechanisms play several important mnemonic roles in a plastic recurrent neural network. Thereby, it challenges the traditional view that memories are encoded solely as a strengthening of synaptic weights. Instead, part of the memory is encoded in the nucleus itself, through epigenetic alterations. These results thus serve as a springboard for further computational and theoretical predictions on how memory processes are affected by epigenetic changes.

We have shown that an epigenetic-based upregulation of IE and synaptic plasticity can compensate for memory loss associated with molecular turnover, synaptic noise and synaptic loss. These results are consistent with experimental studies highlighting the importance of epigenetic mechanisms in maintaining memory in healthy conditions,^16,19,22^ and with their link to cognitive decline when gone awry.^55,56^ Nevertheless, our model generalizes multiple epigenetic mechanisms, such as DNA methylation or histone acetylation, into a single variable that we name ‘epigenetic priming’. In reality, epigenetic plasticity is diverse and multi-faceted in nature. In addition to the well-established neuronal histone modifications of acetylation, phosphorylation and methylation,^57^ recent studies have also identified that histones can be dopaminylated^58^ or serotonylated,^59^ which provides an intriguing link to neurotransmitter signalling directly affecting the epigenome. In like manner, neuronal DNA can not only be methylated,^60^ but also hydroxymethylated,^61^ both of which impact on synaptic plasticity-related gene expression.^62,63^ Multiple such epigenetic priming variables should thus be considered in future models, either individually or in combination,^17,64^ to apprehend the full complexity of mnemonic epigenetic codes. Considering that each nucleus contains ∼30 000 nucleosomes, each composed of eight core histone proteins, each of which harbours ∼30 modifiable amino acids, this poses a formidable computational challenge. Intriguingly, however, it is precisely this high complexity of epigenetic modifications that makes this model also appealing with respect to incorporating synapse-specific stimuli, because from a purely computational point of view, each synaptic weight can, in principle, be encoded by a given epigenetic code.

In our model, we considered epigenetic priming as stable during the entire duration of the simulation, and especially over multiple reactivations. Experimentally, epigenetic priming of engram cells in mice has been shown to last for ≤5 days following learning,^26^ and that of excitatory neurons for ≤30 days post-encoding.^24^ However, it could, in principle, last over a lifetime, as it does for early-life stress-induced epigenetic markers in neurons^65^ and for disease-associated cellular memories following inflammation in astrocytes.^66^ Whether and how such stability in memory-bearing cell assemblies is achieved remains experimentally and computationally unclear but is also of great interest in the field of cellular epigenetic memories in developmental differentiation.^67,68^ There, three factors have been identified computationally as crucial for the persistence of epigenetic memories^67^: (i) there is a substantial density difference between chromatin compartments; (ii) epigenetic marks spread in three dimensions thanks to the interplay between ‘reader’ and ‘writer’ enzymes; and (iii) epigenetic enzymes are limited in abundance relative to their histone substrates. In postmitotic engram neurons, all three conditions are met: (i) chromatin compartmentalization varies within engram nuclei^26,36,69^; (ii) both ‘reader’ (that recognize epigenetic marks) and ‘writer’ (that put the epigenetic marks in place) enzymes are at play^25,30,36^; and (iii) these enzymes are, by default, limited in comparison to histone proteins. Nevertheless, further work also needs to combine cell-intrinsic epigenetic priming with existing models of long-term memory consolidation.^70-72^ Given that memory consolidation involves multiple brain areas, it would be particularly interesting to decipher brain region-specific epigenetic effects and whether and the extent to which they vary spatiotemporally.

Of note, in our model synaptic recurrent weights are still needed for information encoding. As such, they highlight an intricate interplay between synapse-based and chromatin-templated plasticity. Epigenetic priming serves as a complementary mechanism that helps to maintain memories across time and differs from a direct regulation of excitability and plasticity-related proteins. In other words, epigenetics acts as a secondary or ‘meta-learning’ process that gates the network dynamics only for learning-activated neurons. For example, epigenetic priming does not seem to bias allocation *a priori* unless it involves a direct increase in excitability (Fig. 2). This is reminiscent of a study showing that pharmacological epigenetic priming primarily reduces the threshold for neural activity to trigger transcription and does not, *per se*, affect the dynamics of the neuron, such as its firing.^73^

Nevertheless, one could also envision a framework in which, once a memory trace is established, sustained synaptic modifications are no longer necessary for its long-term maintenance (although they remain essential for its subsequent recall). Supporting this idea, studies by Ryan *et al*.^74^ in the mouse and by Chen *et al*.^75^ in *Aplysia* have shown that memories can be recovered even after post-training disruption of synaptic plasticity, either through inhibition of protein synthesis or through reconsolidation-based erasure of learning-induced synaptic changes.

Regarding memory selectivity, our model predicts that the silent nature of epigenetic memory retention allows for protection of ensembles from interfering. In contrast, a direct increase of excitability and plasticity-related proteins would lead to memory interference (Fig. 4), which corresponds to experimental studies showing that learning-induced increase of excitability leads to memory linking.^51-53^ To explain this, we speculate that epigenetic priming is more energetically efficient than, for example, continuously sustaining plasticity such as IE,^76-78^ although it should be noted that epigenetic priming also comes with a metabolic cost.^79,80^

Lastly, the present model focuses solely on the memory engram paradigm, and other cell types and tasks might require more complex weight profiles. Further work would also be needed to understand how epigenetic mechanisms (and other non-synaptic mechanisms; Supplementary Box 1) could help in maintaining more complex forms of memory or lead to aberrant memory performance in various neurological disorders.

These limitations notwithstanding, this study constitutes the first endeavour to integrate epigenetic mechanisms in computational models of memory and finds that epigenetic plasticity can support memory allocation, maintenance and specificity, in addition to counteracting memory loss attributable to synaptic turnover.

## Supplementary Material

awag094_Supplementary_Data
