# Supplementary material for: A computational framework for epigenetic plasticity in memory
Source: Brain. 2026 Mar 7;149(6):1885–92. doi: 10.1093/brain/awag094 (PMC13232041; doi:10.1093/brain/awag094)
Supplement: awag094_Supplementary_Data [file awag094_Supplementary_Data.pdf]

Supplementary Information

Epigenetic mechanisms can rescue memory loss due to reduced maximum weight

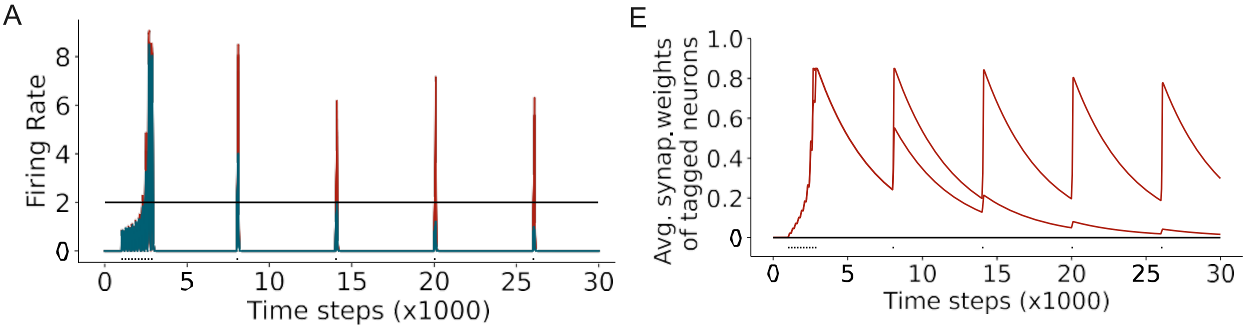

Epigenetic priming can rescue memory loss due to faster synaptic decay

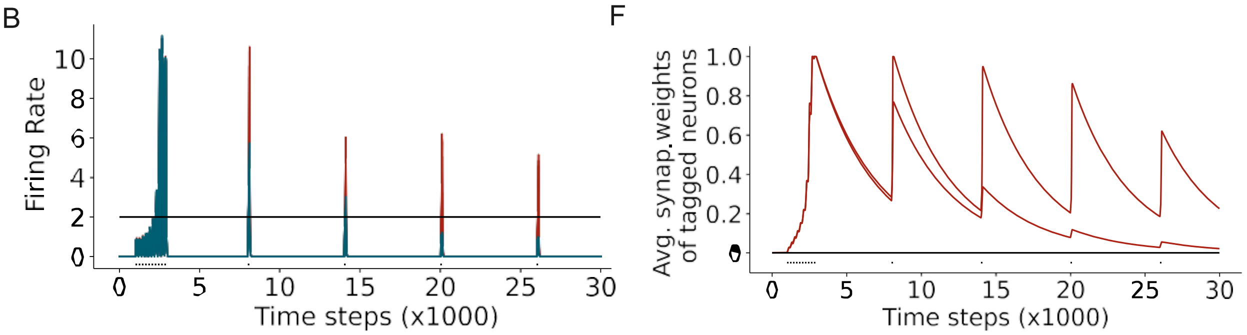

Epigenetic priming can rescue memory loss due to synaptic noise

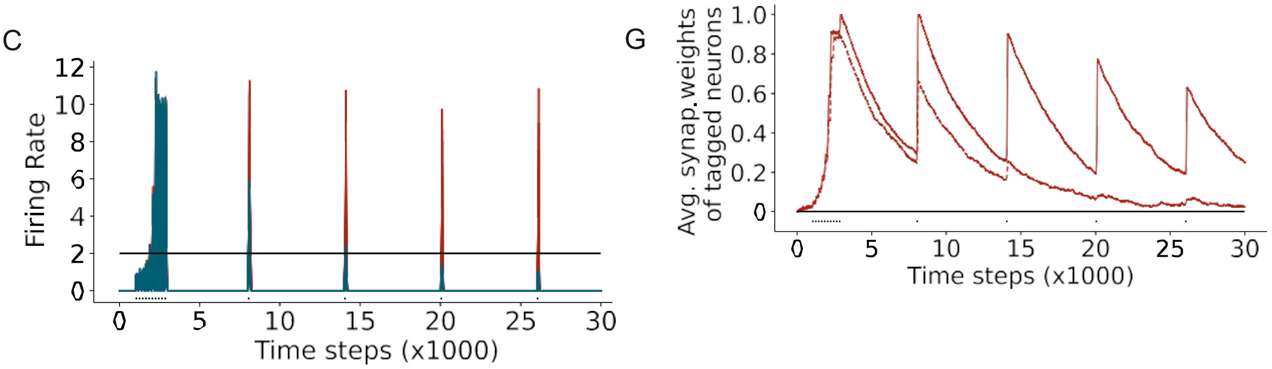

Epigenetic priming can rescue memory loss due to synaptic pruning

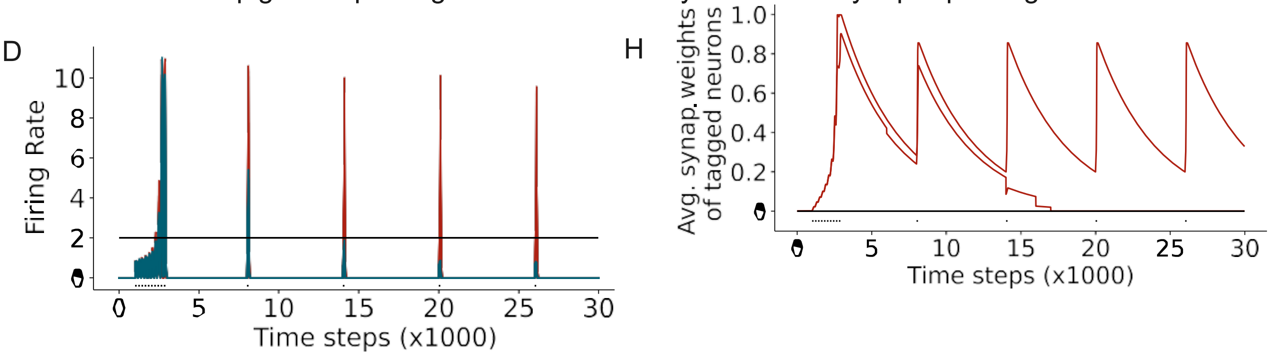

Supplementary Figure 1: Epigenetic mechanisms help to prevent memory degradation due to various biological processes. a-d) Firing rate of neurons across time without (in green) and with epigenetics (in red) under reduced maximum weight (in a), faster synaptic decay (in b), noisy synaptic update (in c), and synaptic pruning of weak synapses (in d). In all conditions, an ensemble is formed by initial stimulation of subsets of neurons (Methods), firing rates of neurons from these ensembles decrease across time falling below the active threshold (dashed line) indicating memory loss. However, epigenetic mechanisms can rescue this loss of memory in each condition. Black bars represent stimulation of the network, the first one corresponding to encoding and the last four to recall. e-h) Averaged synaptic weights of neurons tagged during encoding across time when epigenetic modifications are considered (solid line) and when not (dashed line) under the different biological mechanisms that can lead to memory loss. The synaptic weights are recused by the epigenetic modifications without which they decrease to 0.

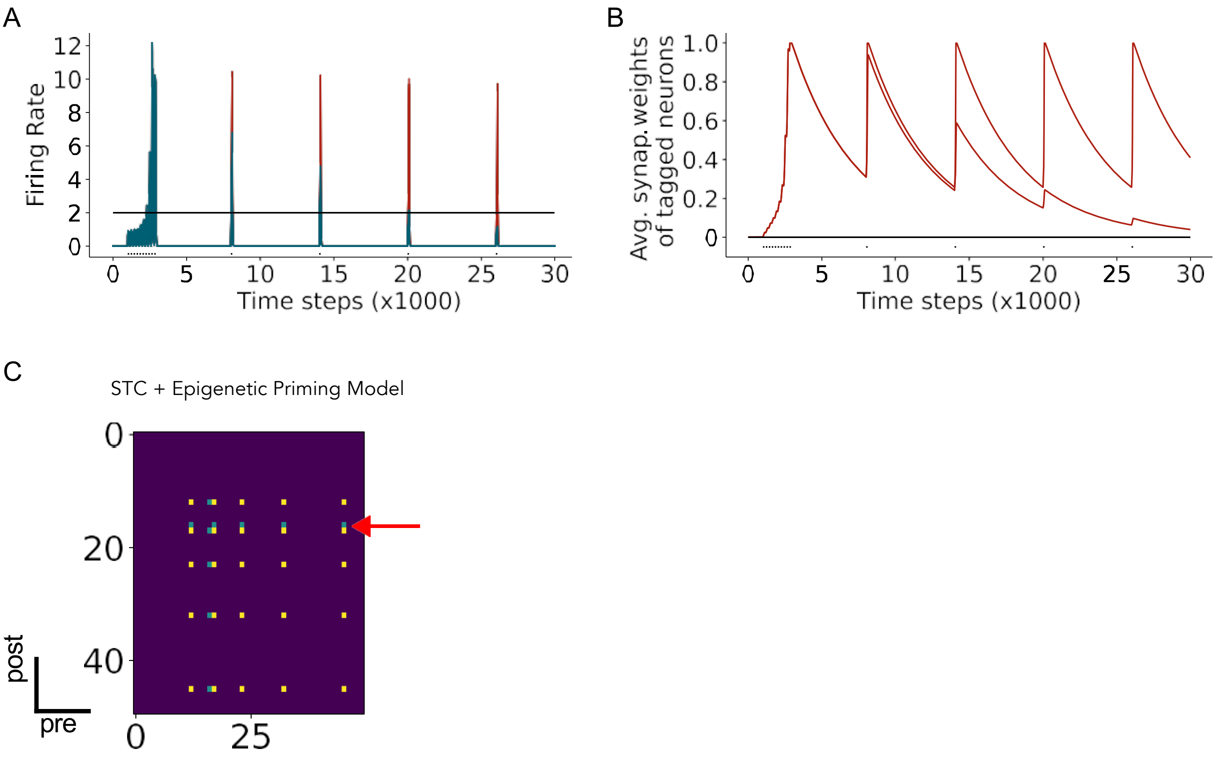

Supplementary Figure 2: Synaptic Tag and Capture processes in the absence of epigenetic mechanism cannot rescue memory loss. a) Firing rate of neurons across time without (in green) and with epigenetics (in red) in our model of synaptic tag and capture plasticity. In both conditions, we observe ensemble formation after initial stimulation of subsets of neurons (Methods), firing rates of neurons from these ensembles decrease across time falling below the active threshold (dashed line) indicating memory loss. Although the weight decay is slower compared to Fig1 and connection with synaptic tagged decay slower. However, epigenetic mechanisms can rescue this loss of memory in each condition. Black bars represent stimulation of the network, the first one corresponding to encoding and the last four to recall. g) Averaged synaptic weights of neurons tagged during encoding across time when epigenetic modifications are considered (solid line) and when not (dashed line) under the different biological mechanisms that can lead to memory loss. The synaptic weights are recused by the epigenetic modifications without which they decrease to 0. c) Matrix of all connections based on whether they were synaptically tagged (in green) and synaptically tagged + epigenetic priming (in yellow) or none (in magenta). Not all connections that underwent synaptic tagging received epigenetic priming (red arrow), highlighting the importance of epigenetic mechanisms for long-term memory storage.

Box 1 Other cellular processes in need of computational modelling for memory

Beyond epigenetic mechanisms, the following intra- and extracellular processes also need to find their way into computational models of memory:

Genetic mechanisms.[81] Both learning[82] and exploring a novel environment83 produce double-stranded DNA breaks in neuronal cells, which are necessary for immediate early gene transcription. Together with documented cases of genetic variants of increased memory performance found in the human population[84] these genetic processes need to be addressed in computational models of memory.

Gene expression changes. Gene expression patterns are a fundamental pillar of cellular identities, and multiple studies have shown their transcriptional importance for memory. Jointly, gene expression changes define gene regulatory networks, the mnemonic modelling of which has already started.[85,86]

Self-perpetuating protein assemblies. Once triggered, abnormally folded proteins form a molecular sink for further malformation and are a cornerstone of proteinopathies leading to neurodegeneration.[87] In like manner, and as speculated early on[12], proteins might constitute self-perpetuating mnemonic scaffolds, and at least two candidates, cytoplasmic polyadenylation element-binding protein multimerization (CPEB)[88] as well PKMζ-KIBRA dimerization[89] have recently been identified that might fulfil this criterion.

Extracellular mechanisms. Perineuronal nets (PNNs) are extracellular matrix structures surrounding neurons that play a crucial role in synapse stabilization and different memory phases[90], and have recently been suggested as a putative long-term memory storage mechanism.[91] Similarly, cell-to-cell transfer of RNA molecules[86] and virus-like propagation of the immediate early gene *Arc*[92] have also been shown to contribute to memory encoding. Both types of extracellular mechanisms await computational modelling for mnemonic processes.

Supplementary references

81 Gold, A. R. & Glanzman, D. L. The central importance of nuclear mechanisms in the storage of memory. Biochem Biophys Res Commun 564, 103-113 (2021).

82 Madabhushi, R. et al. Activity-Induced DNA Breaks Govern the Expression of Neuronal Early-Response Genes. Cell 161, 1592-1605 (2015).

83 Suberbielle, E. et al. Physiologic brain activity causes DNA double-strand breaks in neurons, with exacerbation by amyloid-beta. Nat Neurosci 16, 613-621 (2013).

84 Papassotiropoulos, A. et al. Common Kibra alleles are associated with human memory performance. Science 314, 475-478 (2006).

85 Benuskova, L. K., N. Computational Neurogenetic Modeling. Springer New York, NY (2007).

86 Gershman, S. J. The molecular memory code and synaptic plasticity: A synthesis. Biosystems 224, 104825 (2023).

87 Brettschneider, J., Del Tredici, K., Lee, V. M. & Trojanowski, J. Q. Spreading of pathology in neurodegenerative diseases: a focus on human studies. Nat Rev Neurosci 16, 109-120 (2015).

88 Si, K., Choi, Y. B., White-Grindley, E., Majumdar, A. & Kandel, E. R. Aplysia CPEB can form prion-like multimers in sensory neurons that contribute to long-term facilitation. Cell 140, 421-435 (2010).

89 Tsokas, P. et al. KIBRA anchoring the action of PKMzeta maintains the persistence of memory. Sci Adv 10, eadl0030 (2024).

90 Gogolla, N., Caroni, P., Luthi, A. & Herry, C. Perineuronal nets protect fear memories from erasure. Science 325, 1258-1261 (2009).

91 Tsien, R. Y. Very long-term memories may be stored in the pattern of holes in the perineuronal net. PNAS 110, 12456-12461 (2013).

92 Sullivan, K. R., Ravens, A., Walker, A. C. & Shepherd, J. D. "Arc - A viral vector of memory and synaptic plasticity". Curr Opin Neurobiol 91, 102979 (2025).
